# Repair of a fistula between the aorta and right ventricular outflow tract secondary to infective endocarditis of a unicuspid aortic valve and previously repaired ventricular septal defect

**DOI:** 10.1186/s13019-024-02746-3

**Published:** 2024-04-16

**Authors:** Rickesh B. Karsan, Katie E. O’Sullivan, Christopher J. Lockhart, Christopher Austin

**Affiliations:** 1 Department of Cardiothoracic Surgery, Royal Victoria Hospital, Belfast, BT12 6BA UK; 2 Department of Cardiology, Royal Victoria Hospital, Belfast, BT12 6BA UK

**Keywords:** Infective endocarditis, Congenital heart disease, Fistula

## Abstract

**Background:**

Infective endocarditis of the aortic valve can result in a wide range of destructive pathology beyond the valve leaflets and annulus which require careful surgical planning to provide appropriate debridement and reconstruction. Failure to do so can result in a failure of surgical treatment, recurrent infection and cardiac failure with concomitant high morbidity and mortality.

**Case report:**

We describe the case of a 45-year-old male with previous patch repair of a ventricular septal defect, who was diagnosed with sub-acute bacterial endocarditis of the native aortic valve and developed a new fistula from the aorta to the right ventricular outflow tract which. This was managed surgically.

**Conclusion:**

This unique case highlights another spectrum of infective endocarditis with a unique approach to repair and management.

## Background

Infective endocarditis (IE) can present with a spectrum of destruction of the fibrous skeleton of the heart. Where there is damage to the fibrous skeleton thorough debridement and reconstruction is required to prevent cardiac failure, recurrent infection, and mortality [1]. Surgery is indicated in cases of left sided IE with severe acute regurgitation, obstruction or fistula resulting in cardiogenic shock; when there is uncontrolled infection with abscess formation, persistent bacteraemia/infection, false aneurysm, enlarging vegetation, prosthetic valve dehiscence, new heart block or fistula; and for prevention of embolic events [2].

Fistulae of the aortic root to right ventricular outflow tract (RVOT) are rare even in the context of IE, with approximately 60% of patients developing heart failure [3]. These result in intra-cardiac shunts which alter haemodynamics. Surgical management of this complex issue is ultimately the standard of care for such patients, with timing and surgical planning essential to reduce peri-operative mortality and morbidity. We present a 45-year-old male with history of perimembranous ventricular septal defect (VSD) repair and echocardiographically-diagnosed bicuspid aortic valve (BAV), found to have native aortic valve endocarditis with the subsequent development of an aorto-RVOT fistulae requiring surgical intervention.

## Case presentation

**Fig. 1:**
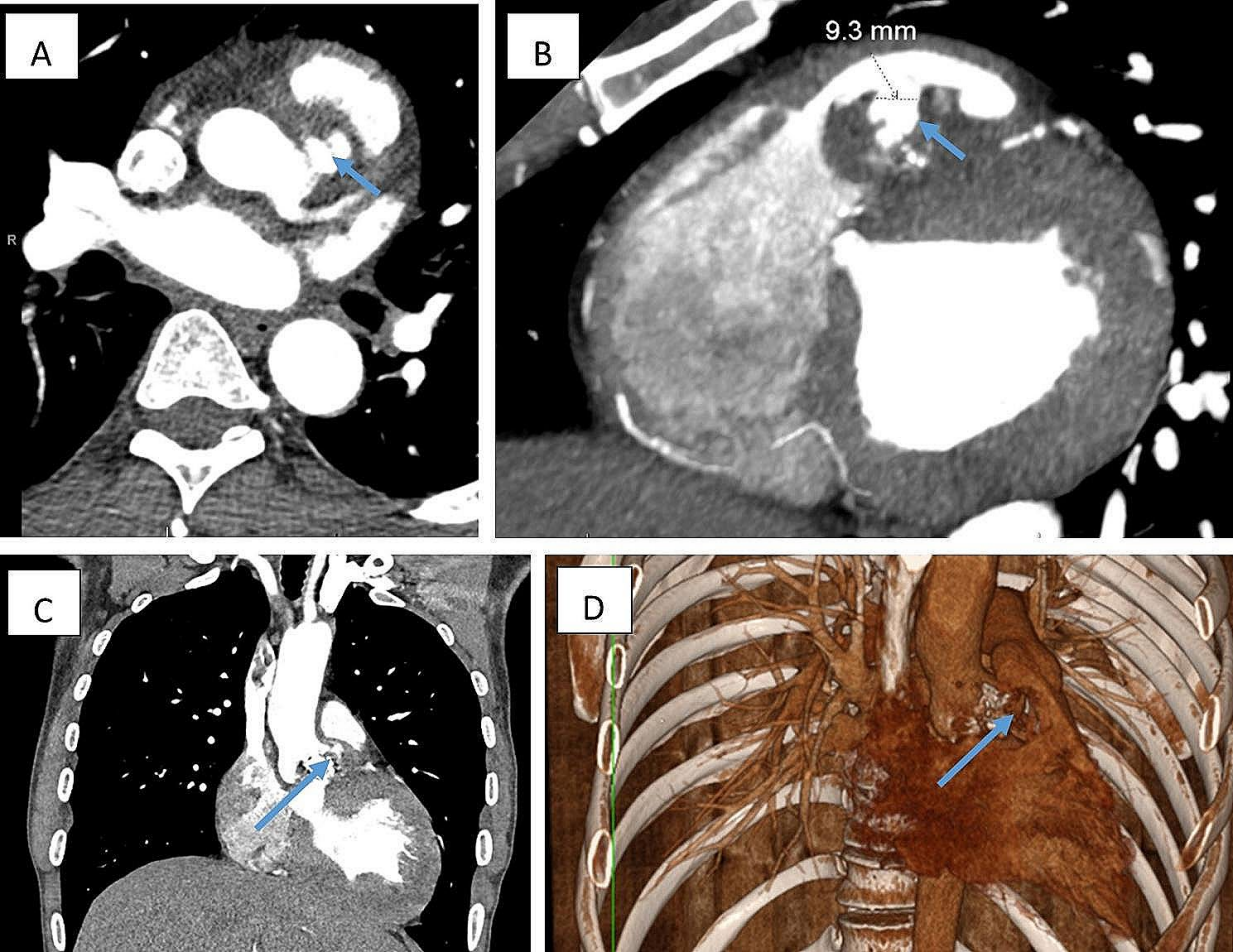
**(A)** Axial CT of the aorta showing fistula arising from the aortic root outpouching towards the right ventricular outflow tract (RVOT), **(B)** CT showing the fistulous communication in the RVOT, **(C)** Coronal CT imaging showing the relationship between the aortic root and the fistula, **(D)** 3D reconstruction showing the fistula

A 45-year-old male with a background of VSD repair as a child and echocardiographically-diagnosed bicuspid aortic valve presented with a four-week history of headaches, malaise, and productive cough. He had been prescribed several antibiotic courses by his general practitioner with no improvement in his symptoms. He was found to have and elevated D-dimer which prompted a computed tomography (CT) pulmonary angiogram. This suggested an abnormal appearance to the aortic valve and convex indentation of the anterior wall of the aortic sinus into the RVOT, which gated CT scan confirmed as a fistula between the aortic root and RVOT (Fig. 1). On examination, a continuous murmur could be heard throughout the precordium on auscultation. A transthoracic echocardiogram (TTE) showed severe aortic stenosis (AS) with heavy calcification. A subsequent trans-oesophageal echocardiogram (TOE) showed a mass attached to the aortic valve (1.6 cm x 1.5 cm). A diagnosis of sub-acute bacterial endocarditis was made, with negative blood cultures and a raised white cell count of 16.1 × 10^9^/L. MRI of the brain was also positive for scattered microhaemorrhages suggesting likely dissemination of infective detritus. The patient was prepared and consented for urgent inpatient surgery.

**Fig. 2:**
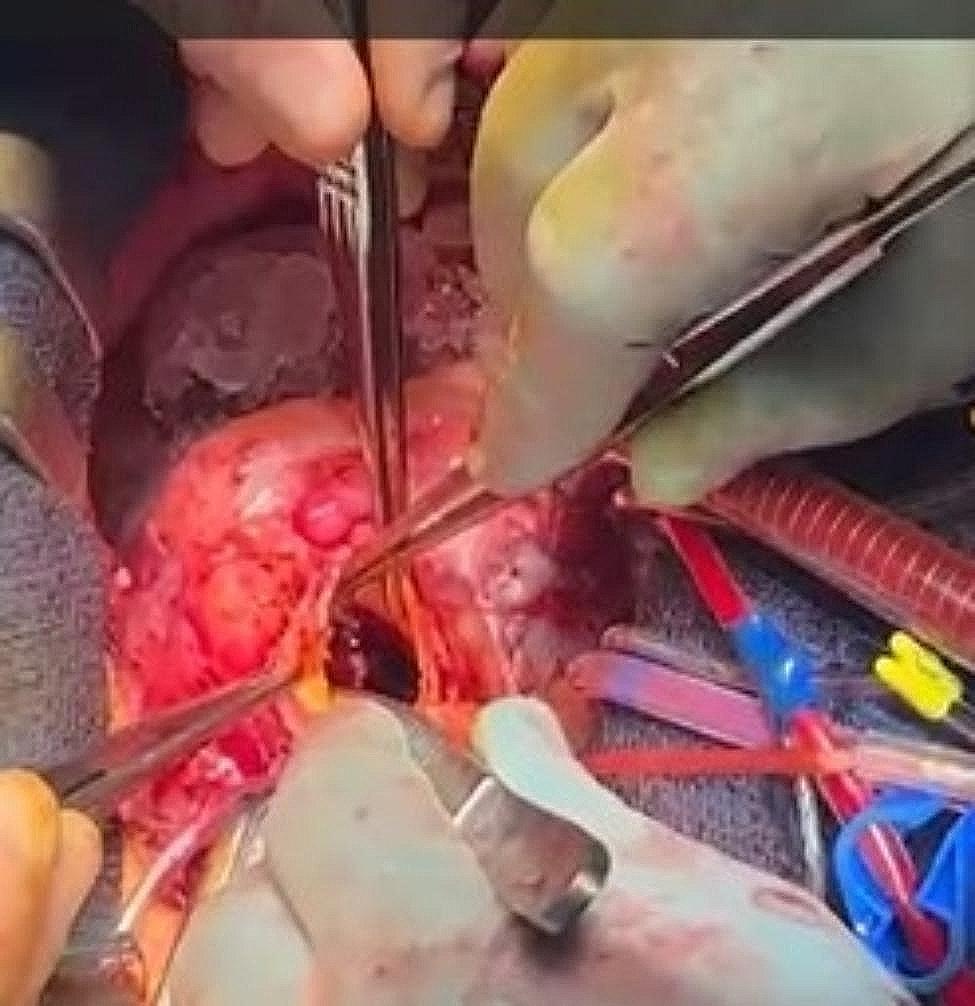
Intraoperative image demonstrating the fistula

Redo-sternotomy was performed and the heart circumferentially freed of adhesions. Heparin was administered and routine central cannulation achieved with insertion of a right superior pulmonary vein vent. Cardiopulmonary bypass was successfully commenced, cross clamp applied and antegrade and retrograde cold blood cardioplegia administered. There was a palpable thrill in the RVOT secondary to the fistula. An oblique aortotomy was performed. The right coronary cusp sinus was eroded with the fistula communicating with the RVOT between the junction of the left and right sinuses, measuring 3.5 cm x 2 cm (Fig. 2): The aortic valve was found to be unicuspid and was extensively calcified, with thrombus and vegetation present.

The native valve was excised, which helped to identify the fistula’s origin at the right coronary sinus with erosion of the aorto-ventricular region. This was extensively debrided of infected and necrotic tissue and washed out. The defect was closed with an oval bovine pericardial patch using a double suture line, initially with interrupted pledgeted 4 − 0 prolene followed by 6 − 0 prolene suture in a continuous fashion circumferentially. Routine aortic valve replacement with a 21 mm mechanical prosthesis in the intra-annular position was completed with a continuous suture technique and the aortotomy closed using a bovine pericardial patch. The cross clamp was removed, and the patient was successfully weaned off cardiopulmonary bypass followed by routine decannulation and closure completed.

**Fig. 3:**
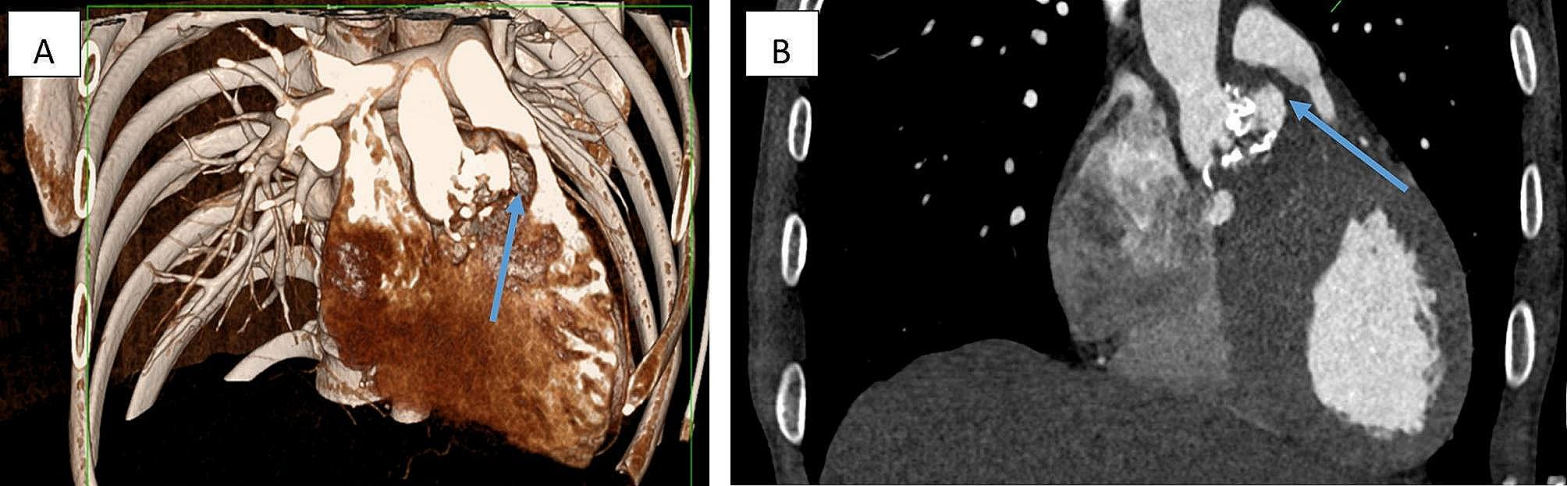
**(A)** 3D reconstruction showing repaired fistula, the remaining bulging was secondary to redundant patch, **(B)** no contrast flow was detected across the repair indicating a competent repair. This was validated by TTE doppler

The postoperative course was uneventful and he was treated with six weeks of antibiotics for staphylococcus sp. which grew from the tissue cultures. Transthoracic echocardiogram and gated CT scan confirmed no residual fistula (Fig. 3).

## Discussion and conclusions

In complex cases of IE where there is damage to the fibrous skeleton, a more individualised approach may be required to facilitate adequate debridement of the infection and reconstruction. IE can occasionally result in destruction of periannular tissue leading to abscess cavities, pseudoaneurysm and potential erosion into adjacent chambers and fistula formation. The clinical presentation of aorto-RVOT fistulae will ultimately depend on the size of the shunt created signs of which may include a continuous murmur and/or thrill [4, 5]. When the infection invades toward the right behind the central fibrous body, it reaches the conduction bundle and the atrioventricular node, and damage to these structures results in heart block [6, 7]. The rate of heart failure, VSD and complete heart block have been shown to be greater in patients with fistula formation. This however, has not been shown to be an independent factor for mortality [8].

Identification of such structural damage secondary to IE through appropriate investigation is beneficial for surgical planning and helps to determine the intra-operative approach to repair with a combination of echocardiography and cardiac CT [9]. They should ultimately be treated in a timely manner surgically to avoid further destruction of cardiac tissue and conduction system. The aim of surgery is to remove infected tissue, clear and debride infection and cavities, remove embolic sources, and restore cardiac integrity and valve function. Various techniques can be employed for aortic valve endocarditis including valve replacement, root replacement, and aortic homograft implantation. A clear long-term advantage of one technique has yet to be proven [10]. Infective extension of aortic valve endocarditis into the fibrous skeleton may warrant replacement or repair of both the aortic and mitral valve, with reconstruction of the intervening the intervalvular fibrosa (“commando” or “hemi-commando” operation).

With native aortic valve IE, valve repair is rarely a surgical option. In cases where the annulus is destroyed, use of a homograft may be beneficial, but a valved conduit can be considered a viable alternative [11, 12]. This is a complex case demonstrates some of the ‘new problems’ that are to be expected and encountered as we continue the long-term follow up of infants and children undergoing complex congenital heart disease surgery. The key finding in this case has been the significant fistulae secondary to SBE on the background of a unicuspid valve with a previous repair of VSD; and the application of the knowledge that we have gleaned from ‘standard aortic work’ over the years and how this applies to cases of complex congenital heart disease. Furthermore, the importance of accurate documentation from previous surgeries cannot be over emphasised as such details can significantly impact the planning of any future red-surgery. As the complex adult congenital heart disease population ages, it is important to address new and more complex problems with creative thinking by using a multi-modality approach.

## Data Availability

No datasets were generated or analysed during the current study.

## Abbreviations

IE: Infective endocarditis
RVOT: Right ventricular outflow tract
VSD: Ventricular septal defect
BAV: Bicuspid aortic valve
CT: Computed tomography
TTE: Tranthoracic echocardiogram
TOE: Trans-oesophageal echocardiogram
